# Multi-omics analysis reveals important role for microbial-derived metabolites from *Botryllus schlosseri* in metal interactions

**DOI:** 10.1128/msystems.00793-25

**Published:** 2025-09-15

**Authors:** Dulce G. Guillén Matus, Caroline M. Donaghy, Nidhi Vijayan, Zachary T. Lane, Matthew Howell, George G. Glavin, Alfredo M. Angeles-Boza, Spencer V. Nyholm, Marcy J. Balunas

**Affiliations:** 1 Department of Microbiology and Immunology, University of Michigan 1259, Ann Arbor, Michigan, USA; 2 Department of Chemistry, University of Connecticut 7712, Storrs, Connecticut, USA; 3 Department of Molecular and Cell Biology, University of Connecticut 7712, Storrs, Connecticut, USA; 4 Department of Pharmaceutical Sciences, University of Connecticut 242849, Storrs, Connecticut, USA; 5 Saint-Gobain Research North America, Northborough, Massachusetts, USA; 6 Institute of Materials Science, University of Connecticut 7712, Storrs, Connecticut, USA; 7 Department of Medicinal Chemistry, University of Michigan 1259, Ann Arbor, Michigan, USA; Stellenbosch University, Stellenbosch, South Africa

**Keywords:** marine tunicates, *Botryllus schlosseri*, metallome, microbiome, metabolome and pan-metabolome, multi-omics data integration, host-microbe-metal interactions

## Abstract

Marine microbial communities govern many of the biological and chemical processes in the ocean, including element cycles, ecosystem health, and disease. Marine organisms are surrounded by microbes, with complex molecular interactions occurring between bacterial symbionts, eukaryotic hosts, and their pathogens or prey. Trace metals in the ocean can be either beneficial or detrimental to marine life depending on their concentrations and bioavailability. Multiple marine tunicate species are known to bioaccumulate trace metals in their mantel, and research suggests that tunicate microbiota plays an important role in this process. *Botryllus schlosseri*, a marine colonial tunicate, has become a model organism for cellular and developmental studies, yet its ecological interactions are still not well understood. Using an integrated multidisciplinary approach, we established a comprehensive baseline and explored correlations between members of the *B. schlosseri* microbiome, metabolome, and metallome to elucidate the ecological effects of trace metals in host-microbe-pathogen interactions. We identified significant correlations between metals, including manganese, nickel, cerium, zinc, and cobalt, with various metabolites and bacterial taxa. These findings offer insights into *B. schlosseri*’s biological and chemical interactions with microorganisms and their environment, contributing to bridging the knowledge gap of host-microbiome-environment interactions and establishing a foundation for continuing research on the ecological effects of trace metals in these biological systems.

**IMPORTANCE:**

Given the importance of marine invertebrates and their microbial communities in marine ecosystems, we sought to characterize the largely unknown microbial associates, metal sequestration, and metabolite production of the marine colonial tunicate, *Botryllus schlosseri*, a model organism for cellular and developmental studies. Using an integrated multidisciplinary approach, we identified significant correlations between metals, metabolites, and bacterial taxa. *B. schlosseri* tissue was highly enriched in metals compared to seawater, and *B. schlosseri* microbiome beta-diversity was significantly different from seawater. We also introduced the concept of the pan-metabolome to classify metabolites based on their presence or absence across complex samples and found microbial metabolites in both the core and flexible metabolome. These findings offer insights into *B. schlosseri*’s biological and chemical interactions with microorganisms and their environment, bridging the knowledge gap of host-microbiome-environment interactions and establishing a foundation for continuing research on the ecological effects of trace metals in these biological systems.

## INTRODUCTION

Metal ions in marine environments play a pivotal role, having both positive and negative effects on marine ecosystems, while influencing ecological processes and contributing to the overall health of these ecosystems. About a dozen elements with atomic mass above 50 are known to have biological roles, often as cofactors, as promoters of redox chemistry, or as structural elements in proteins (1, 2). Iron, manganese, nickel, zinc, cobalt, and vanadium have been the most characterized in oceanography (3). Iron, probably the most well studied, is a limiting nutrient for many biological processes and plays an essential role in primary production (3). Iron is also typically found at high concentrations in planktonic communities (4). However, although the biochemical functions of many metal ions are extensive, we currently lack a complete understanding of the role played by these important trace elements, especially in complex marine environments (3, 5). It has been demonstrated that small changes in metal concentrations, as low as sub-nanomolar, can have drastic effects in marine environments (6). Coastal environments, enriched in many trace metals, exhibit a remarkable biological diversity, in part facilitated by the interface between terrestrial and oceanic influences, which fosters a unique and vital habitat for a wide array of marine species of microbes and complex organisms (7, 8).

Microbial communities in the ocean govern many processes, including primary production, ecosystem health and disease, and many element cycles (9). They are found in planktonic states in the water column, forming biofilms on solid surfaces, and/or inhabiting macroorganisms (10). Interactions between microorganisms and animals can range from the broad categories of mutualism, in which both parties benefit, to pathogenic relationships, in which one or more parties are harmed. Marine invertebrates, especially filter feeders, comprise the largest biodiversity of multicellular eukaryotes in the ocean and harbor complex relationships with microbial communities (11, 12). Many marine invertebrates enter into beneficial microbial symbioses that allow them to make use of habitats that would otherwise be unavailable to them (e.g., tropical corals and hydrothermal vent tubeworms) (13–15).

The study of host-associated microbial communities is crucial for understanding animal physiology, especially in relation to pivotal roles for microbiota in host development, nutrition, and behavior (16, 17). However, more recently, microbiome research has often been paired with metabolomics analyses to further understand how host chemical output shapes their microbiota and how microbially derived molecules influence host physiology (17, 18). While genomics (e.g., 16S rRNA community profiles) provides insight into the microbial taxa present in the host, metabolomics provides a chemical fingerprint of cellular processes occurring within and between hosts and microbes (19, 20). Current comparative metabolomics methods allow for rapid analysis of large number of samples, facilitating chemical ecology investigations across host species and ecosystems, including their complex associations with microbial communities.

Tunicates, classified under the phylum Chordata, are filter-feeding invertebrates known to harbor bacteria in their digestive tracts, tunics (21), and throughout their bodies (22). In some cases, tunicate-associated bacteria have been shown to be symbiotic or mutualistic with the host, such as defending against pathogenic microbes, acting as feeding deterrents, and/or helping with nutrient acquisition, thus making them vital for host survival (23, 24). Tunicates have also emerged as noteworthy sources of natural products, known for their valuable metabolites with antimicrobial, antiparasitic, and anticancer properties (24–27). Tunicates are also important for marine food webs, triggering biogeochemical flux from the surface to deep waters, and have even been linked to being potential environmental stress indicators (28), including as bioindicators for metals in marine ecosystems (1, 29, 30).

The colonial tunicate, *Botryllus schlosseri*, is a cosmopolitan species that inhabits shallow intertidal marine habitats around the world (31). *B. schlosseri* has emerged as a prominent model organism in several areas of biology, including studies of histocompatibility, self/non-self recognition, stem cell biology, and development (32–35). However, little is known about the microbiome of *B. schlosseri* with even more limited studies of the metallome or metabolome of this species. Two microbiome studies of this and other tunicates found their microbial communities to be conserved within species, suggesting that the mucosal layer of the pharynx may harbor specific symbionts (31, 36). Thus, although there has been emphasis on developing *B. schlosseri* as a model organism, there are still significant gaps in the understanding of its ecology and environmental adaptations.

Using a multidisciplinary approach, we investigated the *B. schlosseri* microbiome, metabolome, and metallome, including multi-omics integration, to determine the importance of microbes, metabolites, and metals in *B. schlosseri* communities. Our integrative multi-omics approach provides a novel perspective regarding on how these tunicates flourish in their environment, aiming to further elucidate the importance of microbes, metabolites, and metals in these marine systems. Our findings provide the first steps for understanding ecological effects of trace metals in the host-microbe interactions of *B. schlosseri*. Studying the intricate relationship between *B. schlosseri*’s microbiome, metabolome, and metallome helps to unravel the complex interplay between these organisms and their environment, providing crucial insights into the ecological dynamics, adaptive strategies, and potential bioaccumulation effects in marine ecosystems.

## MATERIALS AND METHODS

### Tunicate and seawater sample collection

Eight subtidal *B. schlosseri* colonies, identified via phenotypical characteristics, were collected from artificial substrates via submerged ropes and other structures from three dock sites at Avery Point in Groton, CT (N 41°18′59″, W 72°3′39″). *B. schlosseri* colonies were quickly dipped in 100% ethanol (EtOH) to surface sterilize, rinsed two to three times with sterile artificial seawater (40 g/L Instant Ocean Sea Salts, Blacksburg, VA), and separated into three aliquots—one for each type of analysis. Samples were then placed on ice and transported to the lab where they were stored at −80°C until further processing.

Surface seawater was collected from the same three tunicate collection sites in 5 L quantities. Seawater collections were brought back to the lab and processed immediately. For microbiome and metabolomic analyses, 1 L from each collection was vacuum filtered in triplicate through a sterile vacuum apparatus (Millipore) equipped with 0.22 µm, 47 mm diameter, autoclaved PTFE hydrophobic filter disks (Millipore) to obtain samples for each type of analysis. Filter disks were removed from the apparatus and stored at −80°C until analysis. For metal analyses, 1 L of seawater was transferred to ICP digiprep tubes (SCP Science, DigiTUBES) and kept in a cooler during transport to the lab. The seawater was filtered with a sterile 0.22 µm filter (ThermoScientific, Nalgene Prefilter Plus), preserved with trace metal grade HCl, and stored at 10°C until analysis.

### Metallomics

#### Metal quantification using ICP

Metal analyses were conducted using *B. schlosseri* or seawater samples. Quality control samples (i.e., laboratory reagent blank, laboratory fortified blank, laboratory fortified matrix, standard reference matrix, and calibration standards) were utilized for all experiments and prepared using the same digestion matrices as the samples. Calibration standards for tunicate samples were prepared using the multi-element standard solution ICP Stock Standard and ICP-MS Memory Check Solution B (High Purity Standards, Charleston, SC). For seawater samples, calibration standards were prepared using the multi-element standard solution QC1 (Inorganic Ventures Labs, Christiansburg, VA). The acid matrix for digestion of tunicate samples consisted of NanoPure water (filtered using a Barnstead NANOpure Diamond filtration system with a 0.2 µm pore size filter), HNO_3_, and H_2_O_2_. For seawater samples, the acid matrix consisted of HNO_3_ and HCl. Quality controls and calibration standards for seawater samples used Instant Ocean (Instant Ocean, Blacksburg, VA), prepared at a concentration of 16.9089 g/L with NanoPure water. Analytical precision was measured as a relative standard deviation that was consistently below 5%.

#### Metal analysis of *B. schlosseri* samples

Prior to digestion, samples were lyophilized overnight using a FreeZone Freeze Dryer (Labconco Corporation, Kansas City, MO) and homogenized using an agate mortar and pestle. Most samples were analyzed individually with the exception of the “mixed” sample which consisted of four individual tunicate samples that were homogenized together in order to evaluate metal concentration yields from a larger sample. Tunicates were digested following modified EPA protocols 200.3 (37). Each sample was refluxed in trace metal grade HNO_3_ for 1 h at 92–95°C using a hot block (Environmental Express, Charleston, SC). Once samples were cooled, 0.3 mL of NanoPure water and 0.75 mL of trace metal grade H_2_O_2_ were added, and samples were reheated until bubbling subsided. Samples were then cooled and brought to a final volume of 15 mL with NanoPure water. Filtering was unnecessary due to complete digestion, as indicated by lack of precipitants in solution. Samples were analyzed using inductively coupled plasma mass spectrometry (ICP-MS; Perkin-Elmer ELAN/DRC-e, Shelton, CT) following EPA analysis method 6020A (38) to determine metal concentrations of vanadium (V), manganese (Mn), iron (Fe), cobalt (Co), nickel (Ni), copper (Cu), zinc (Zn), and cerium (Ce). The limits of detection (LODs) were determined to be below 0.2 ppm for each metal, except for iron and zinc, which had LODs below 15 and 3 ppm, respectively (Table S1).

#### Metal analysis of seawater samples

Seawater samples were filtered with a 0.20 µm Nalgene Prefilter Plus GFP + 0.2 µm CA filter (Thermo Scientific, Waltham, MA), preserved with trace metal grade HNO_3_, and stored at 4°C until digestion. Seawater samples were digested following EPA protocols 200.7 (39). Preserved seawater samples were individually transferred to new digestion tubes in 25 mL increments, to which 0.25 mL trace metal grade HNO_3_ and 0.125 mL of trace metal grade HCl were added, then refluxed at 92–95°C within a hot block for 2 h or until the samples had reduced in volume by 12 mL. Samples were then cooled and brought to the final volume of 25 mL using NanoPure water. Processed seawater samples were analyzed using an ICP-Optical Emission Spectrometer (ICP-OES; Spectro Arcos ICP-OES, SPECTRO Analytical Instruments GmbH, Kleve, Germany) to determine metal concentrations of V, Mn, Fe, Co, Ni, Cu, Zn, and Ce via true axial plasma observation. The LODs for metals in the seawater samples were determined to be equal to or less than 0.002 ppm (Table S1).

#### Statistical analysis

Bioaccumulation results for tunicate samples were calculated via fold increase analysis, dividing the average metal value within each tunicate sample by the average metal value found in the surrounding seawater. Error corresponding to metal accumulation was calculated using error propagation since the *B. schlosseri* and seawater samples were derived from different populations. After averaging individual metal concentrations for both *B. schlosseri* and seawater samples, the corresponding average and standard deviation values were applied to equation 1, where *z* corresponds to accumulation value, while *x* and *y* represent the average water and tunicate samples. All statistical analyses for the metal data were performed in Microsoft Excel using two-tailed, homoscedastic *t*-test analysis.


(1) $$\Delta z=z\sqrt{\frac{{\Delta x}^{2}}{x}+\frac{{\Delta y}^{2}}{y}}$$

### Bacterial community analysis

#### DNA extraction and 16S rRNA analysis

Tunicate samples and seawater filters were thawed, bead-beated with Fastprep-24^TM^ (MP Biomedicals, Santa Ana, CA) at 4.5 m/s for 45 s, centrifuged at 16,000 × *g* for 1 min, and the supernatants were subjected to DNA extractions. Two replicates of negative controls, i.e., tubes without tissues, were processed alongside all samples. DNA was extracted using Qiagen PowerMag soil DNA isolation kits (27100-4-EP). The V4 region of the 16S rRNA gene was amplified via PCR using primers 515F (GTGYCAGCMGCCGCGGTAA) and 806R (GGACTACNVGGGTWTCTAAT). PCR conditions included an initial denaturation at 95°C for 3 min, followed by 30 cycles of 95°C for 30 s, 55°C for 30 s, and 72°C for 1 min and 30 s, with a final extension at 72°C for 10 min. PCR products were quantified using Qubit, purified with the QIAquick PCR Purification Kit, and sequenced using the Illumina MiSeq platform with the MiSeq Reagent Kit v2 (2 × 250 bp) at the University of Connecticut Microbial Analysis, Resources, and Services (MARS) facility. Sequences were deposited in the National Center for Biotechnology Information (NCBI) Sequence Read Archive (SRA) (accession number: PRJNA1039848).

Raw sequences were denoised and demultiplexed using QIIME 2.2020 (40), and amplicon sequence variants (ASVs) were generated using DADA2 (41). Sequences were aligned against a naïve-Bayes trained Greengenes reference database (V2.2022) at a 99% or higher identity to the reference sequences (42). Sequences assigned as chloroplast or unassigned at kingdom level were removed. Data processed with QIIME2 were converted to phyloseq objects using qiime2R (v0.99.6). The final ASV-abundance matrix was normalized with transform_sample_counts to the minimum number of reads in the sample set (6933). We also rarefied the data using rarefy_even_depth and compared the statistical data with normalization, finding that both methods generated similar results. Beta diversities were obtained using the Bray-Curtis (43) and Weighted UniFrac (44) modules in QIIME2 and plotted in R using the phyloseq package. Alpha diversities, including observed, Shannon, and inverse Simpson indices, were calculated with the estimate_richness function from the phyloseq package.

### Metabolomics

#### General experimental

All solvents for LC-MS/MS were LC-MS grade from Sigma-Aldrich (St. Louis, MO). Solvents for extraction were HPLC grade from Sigma-Aldrich (St. Louis, MO).

#### *B. schlosseri* and seawater extraction for mass spectrometry (MS) metabolomics

Tunicate samples or seawater filters were thawed at room temperature, and all extraction glassware, containers, and tools were pre-rinsed with extraction solvents. Samples were transferred to glass beakers and extracted four times with 2:1 dichloromethane:methanol (DCM:MeOH; ~5–10 mL), including maceration, stirring, and filtering to afford an exhaustive extraction. Extracts were then concentrated via rotary evaporation at 38°C using a Büchi rotavapor model R-210 (New Castle, DE, USA), transferred to vials, and stored at −80°C.

#### MS data acquisition and processing

Extracts were prepared at 1 mg/mL in MeOH. Data were acquired using a Bruker timsTOF Pro2 coupled to an ELUTE UPLC (Bruker-Daltonics, Billerica, MA, USA). Each sample (2 µL) was injected in technical triplicate using a Bruker Intensity Solo 2 C_18_ column (100 × 2.1 mm, 1.8 µm). Samples were eluted using a 0.3 mL/min gradient of mobile phases A (0.1% formic acid in water [H_2_O]) and B (0.1% formic acid in acetonitrile [ACN]) beginning at 5% B for 0.5 min. The gradient was then increased linearly from 5% to 40% B over 3.5 min, from 40% to 98% B over 4.0 min, held at 98% B for 1.0 min, returned to 5% B for 0.5 min, and finally held at 0.5% B for 1.0 min.

Data acquisition was performed in positive ionization mode using a VIP-HESI (Vacuum Insulated Probe Heated Electrospray Ionization) source, with a collision energy of 10 eV, capillary voltage of 4,500 V, dry temperature of 220°C, sheath gas temperature of 400°C, mass range of 150–2,200 *m/z*, mobility (1/*K*_0_) range of 0.55–1.90 V·s/cm^2^, and ramp time of 100 ms. Fragmentation data were acquired with a collision energy of 50 eV, using two PASEF MS/MS scans per cycle, with active exclusion release after 0.1 min, for a total cycle of 0.53 s. 

Once acquired, MS data were preprocessed using Bruker MetaboScape version 9.0.1 (Bruker-Daltonics, Billerica, MA, USA) using the MCube T-Rex 4D Metabolomics workflow for peak picking and alignment. The intensity threshold was experimentally determined by comparing baseline noise from samples and MeOH blanks, resulting in a threshold of 7,500 counts and a feature table with 2,545 total features before filtering.

The resulting feature table was then processed using the MPACT software (45) with the following parameters: mispicked peak correction—ringing mass window of 0.5 atomic mass units (AMUs), isotope mass window of 0.01 AMU with a maximum isotopic mass shift of 3 AMUs, and a *t_R_* window of 0.05; in-source ion filtering threshold of 0.95 Spearman correlation; median coefficient of variation (CV) of technical replicates of 0.5; and blank filtering using MeOH blanks using a 0.05 threshold. After MPACT filtering, 17.3% of features passed the filtering steps, removing 1,243 blank features, 217 mispicked features, and 281 features found to be nonreproducible across technical replicates.

For *in silico* formula prediction and annotations, MetaboScape, MPACT (45), Sirius (46), NPAtlas (47), and MarinLit (48) were used as annotation databases, with a 10 ppm upper limit for both annotation and formula prediction. When pertinent, annotations were further verified by comparing fragmentation patterns using public data, when available, or using the Competitive Fragmentation Modeling for Metabolite Identification (CFM-ID) spectrum prediction (49). Confidence for each annotation was assessed using Schymanski’s rules for metabolite annotation (Table S5) (50).

#### Metabolomics and multivariate analysis

MPACT (45) was used to conduct statistical comparisons between metabolomes of *B. schlosseri* and seawater using heatmaps, nonmetric multidimensional scaling (NMDS) analyses, and volcano plots. The heatmap was built using normalized raw counts for each feature between minimum and maximum counts for that feature. Samples were grouped on the *x*-axis by overall metabolomic similarity, with features grouped on the *y*-axis. The NMDS plot was generated using Bray-Curtis dissimilarity distance, after averaging technical replicates. The volcano plot was generated using multiple two-tailed *t*-tests, including false discovery rate (FDR) correction using the Benjamini-Hochberg procedure (51).

For the construction of the *B. schlosseri* pan-metabolome, the presence or absence of all features was calculated for every individual. Using group parsing calculations (e.g., references 45, 52), a feature was present if the ratio between its maximum count across all *B. schlosseri* samples and the count from each individual was below 100. A feature was considered absent if its counts were zero or if the maximum over the individual ratio was 100 or higher. To visualize the *B. schlosseri* pan-metabolome, an UpSet plot was generated using the R package UpSetR (53), adding stacked bars to indicate unannotated features as well as the putative origin of any annotated metabolites. According to the information provided in databases used for annotation, each feature was classified as microbial, invertebrate, or mixed origin, when reported in both microbes and invertebrates.

### Multi-omics integration model

Integration of the metabolome, microbiome, and metallome was accomplished using Diablo (Data Integration Analysis for Biomarker discovery using Latent variable approaches for Omics studies [54]), which is an N-integration model from the mixOmics R package (55). A subset of three *B. schlosseri* colonies, for which data across all -omics were available, was utilized for multi-omics integration analysis. Comparison of metals concentrations, OTUs sequence counts, and MS data was facilitated by scaling each data type, beginning with scaling each metal concentration across its own maximum and minimum. Metabolites were normalized using square root and used Pareto scale (56). The OTU sequence counts were normalized using center log ratio (57). Further, any feature with a standard deviation of zero across all data was removed, since these would not contribute to the Diablo covariance model.

To integrate multi-omics data, the N-integration model in Diablo can be run using either full or tuned mode to generate the model. The tuned mode (reduced model) was used for generation of a circos plot based on Spearman’s pairwise correlation coefficients (−1 ≤ *r* ≥ 1) and included only the first and second component of variance from the integration model to reduce complexity and facilitate visualization. To choose the optimal number of components, an M-fold validation was performed with 3 folds and 10 iterations, with the number of components to be tested set at 3. Similarly, the optimal number of features per -omic data set for each of the components (from the previous step) was determined by running an M-fold validation with 3 folds and 10 iterations. Lastly, a hyperparameter of 0.1 was used for the design matrix. The full integration model was used to create a multiblock sparse partial least squares discriminant analysis [(s)PLS-DA] (Fig. S5 and S6) and a correlation network with a hyperparameter of 0.1 for the design matrix and three components. Subsequently, network percolation was performed using the R package associationSubgraph (58) to set up correlation thresholds, facilitating the prioritization of network interactions and enabling easier visualization of correlation clusters. The resulting thresholds for the connections were 0.995 for positive connections and 0.990 for negative connections. Networks were visualized using Cytoscape (59).

## RESULTS AND DISCUSSION

### Metallomics of *B. schlosseri* and surrounding seawater

**Fig 1:**
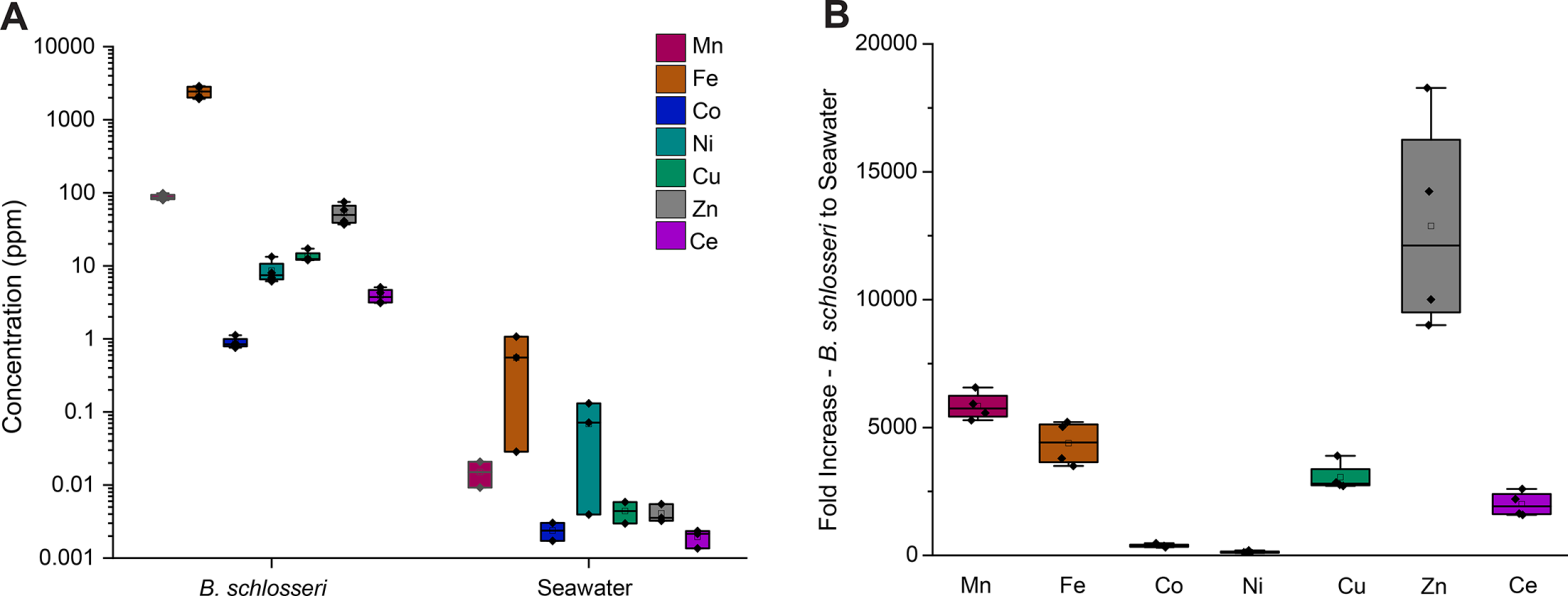
*B. schlosseri* tunicates sequester metals at substantially higher concentrations compared with surrounding seawater. (**A**) Average metal concentrations for *B. schlosseri* and seawater samples in units of parts per million [y-axis on logarithmic scale; vanadium (V), manganese (Mn), iron (Fe), cobalt (Co), nickel (Ni), copper (Cu), zinc (Zn), and cerium (Ce)], indicating substantially higher concentrations of all metals within *B. schlosseri*. Vanadium data not shown for seawater because concentrations were below limit of detection (LOD). (**B**) Fold increase of average *B. schlosseri* metal concentrations relative to those of the surrounding seawater (error bars represent the variation in the fold increases across samples including error propagation).

Using acid digestion and ICP analysis, the average concentrations of vanadium (V), manganese (Mn), iron (Fe), cobalt (Co), nickel (Ni), copper (Cu), zinc (Zn), and cerium (Ce) were determined for both *B. schlosseri* tissues and their surrounding seawater (Fig. 1; Table S2). The concentrations of all measured metals varied relative to one another (Fig. 1A). Iron, manganese, and zinc were the most abundant metals in *B. schlosseri*, while cobalt was the least abundant metal found in the tunicates. The rare earth metal, cerium, was also found to be highly abundant in *B. schlosseri*. In seawater, iron, nickel, and manganese were the most abundant, while cobalt and cerium were the least abundant. To determine the extent to which these metals were accumulated in *B. schlosseri* relative to their environment, we also established fold increases for each metal (Fig. 1B). Concentrations of all measured metals were found to be significantly (*P* < 0.01) higher in *B. schlosseri* than in seawater, especially zinc, manganese, and iron, indicating tunicate metal sequestration.

Our findings are consistent with previous well-established research on the natural abundance of many of these metals—e.g., iron, manganese, zinc, and copper, in seawater (60–63)—and their role as common cofactors in marine biochemistry (64, 65). Zinc was the most substantially increased in *B. schlosseri* versus seawater, which is not surprising given its important role in the structure and function of enzymes involved in gene expression, protein synthesis, and immune function (66, 67). Manganese was the second-most bioaccumulated metal in our study and is known for its roles in energy metabolism and hormone synthesis (68, 69). Iron was also heavily sequestered by *B. schlosseri* and is crucial for many biochemical reactions in biological systems, including electron transport, DNA synthesis, and the regulation of both gene expression and cellular signaling (70, 71). Interestingly, cerium was also bioaccumulated, especially when considering how little cerium was present in the surrounding seawater. Previous studies have found that tunicates sequester substantial quantities of rare earth metals, including cerium (72). This accumulation may indicate that tunicates may exploit unique biochemistry for their proliferation and invasion of habitats. Although the role of cerium in *B. schlosseri* has not yet been elucidated, cerium may aid in antioxidant activity, redox chemistry, detoxification, or structural enhancement of the tunicate tissue (73, 74). Copper was also sequestered by *B. schlosseri*, with previous studies suggesting that copper accumulation may aid in predator deterrence due to the delicate balance between accumulation and toxicity for both tunicates and their larvae (75). The least bioaccumulated metals in *B. schlosseri*, nickel and cobalt, are both heavy metals with low toxicity thresholds but are often found as structural components within enzymes and proteins of biological systems. Although the roles for these metals are not yet defined in *B. schlosseri*, our research provides possible insights into their importance for this colonial tunicate.

### Bacterial diversity of *B. schlosseri* and surrounding seawater

To generate 16S rRNA community profiles, microbial DNA from eight *B. schlosseri* and three seawater samples was amplified and sequenced, resulting in total amplicon reads of 276,709 and 196,968, respectively. The number of amplicon reads for *B. schlosseri* and seawater samples ranged from 4173 to 81,886 and 61,546–69,946, respectively. After normalizing reads from *B. schlosseri* samples to the minimum number of reads, *B. schlosseri* communities were shown to have 2,200 unique taxa, whereas the normalized data set for seawater samples had 2,571 taxa. Plateauing of the rarefaction curves was observed for both *B. schlosseri* and seawater samples (Fig. S1).

**Fig 2:**
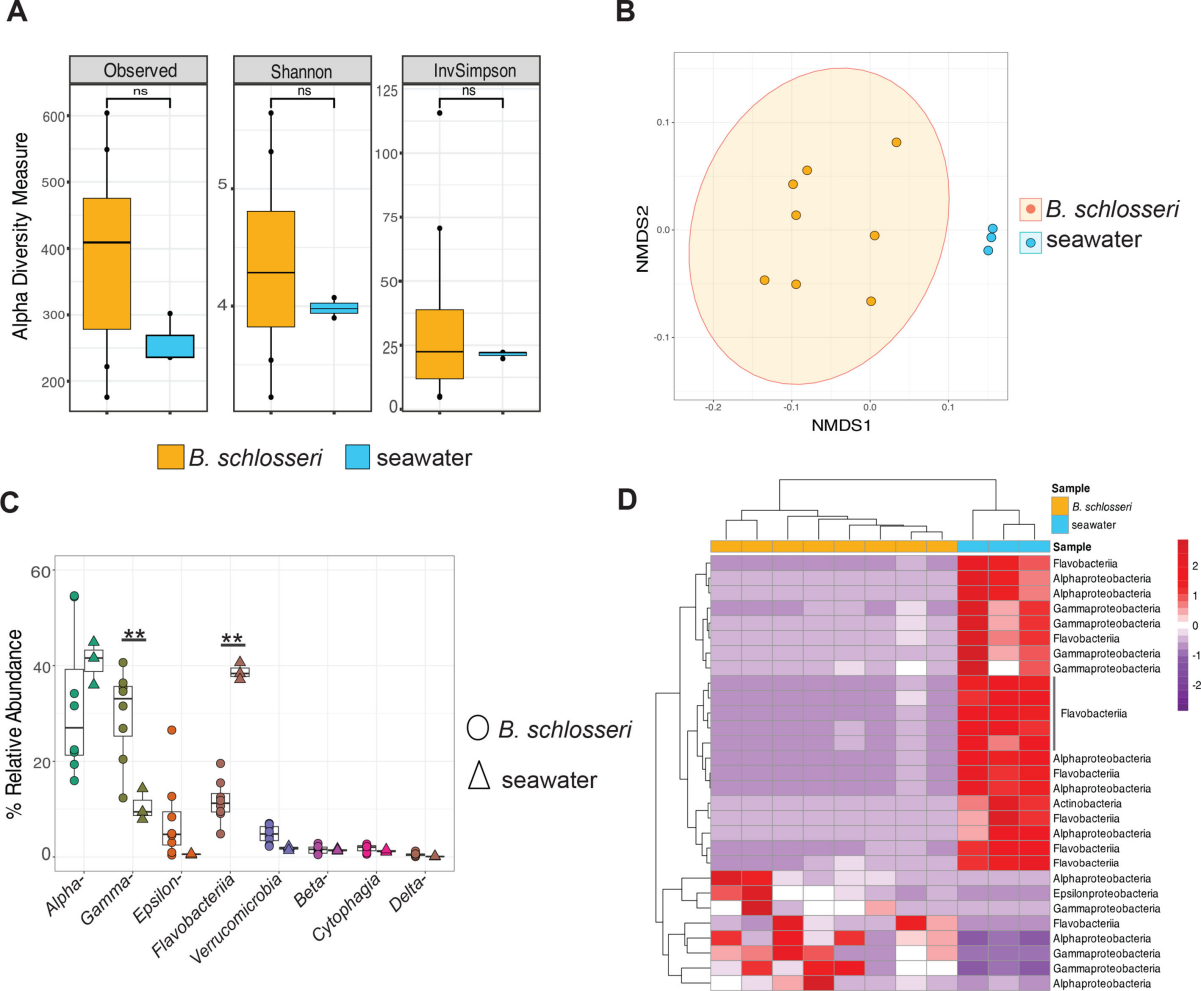
Bacterial diversity in *Botryllus schlosseri* and surrounding seawater. (**A**) No significant difference was found in alpha diversity between *B. schlosseri* and surrounding seawater (*P* > 0.05). (**B**) Nonmetric multidimensional scaling (NMDS) of beta diversity metric, using weighted UniFrac, demonstrated that the bacterial community composition of *B. schlosseri* was distinct from the surrounding seawater. (**C**) Percent relative abundance of bacterial taxa making up >0.1% of the *B. schlosseri* and seawater microbiota indicated that some taxa are more abundant in the tunicate (*t*-test; **, *P*-value < 0.01). (**D**) Heatmap of the most differentially abundant amplicon sequence variants (ASVs) classified to their class taxonomic level.

To consider microbial diversity at the community level, alpha and beta diversity metrics were calculated (Fig. 2A and B). Using the Wilcoxon test, no significant differences (*P* > 0.05) in alpha diversity were observed between *B. schlosseri* and seawater samples (Fig. 2A), possibly due to the fact that tunicates are filter feeders and animals were not depurated before sampling. However, significant differences in beta diversity between tunicate and seawater microbial communities were identified using Weighted UniFrac (PERMANOVA *q*-value = 0.001, pseudo-*F* = 11.82) and Bray-Curtis distance metrics (PERMANOVA *q*-value = 0.001, pseudo-*F* = 4.34). This larger effect size with weighted UniFrac analysis suggests a stronger difference between microbiomes when phylogenetic relatedness is considered, and the smaller effect size with Bray-Curtis suggests differences are less pronounced when only abundance is considered, without accounting evolutionary relationships (43, 44). Further, the differences observed may also be partially attributed to the within-sample variation among replicates of *B. schlosseri* (weighted Unifrac PERMDISP *P*-value = 0.048), although this result is only marginally significant. This suggests that while tunicate-associated microbiomes may exhibit greater heterogeneity compared to seawater communities, additional factors likely contribute to the observed differences in beta diversity.

To further explore the microbial communities of *B. schlosseri* and surrounding seawater, relative abundances of specific taxa were compared (Fig. 2C and D). In *B. schlosseri*, the most abundant classes were Alphaproteobacteria (31% ± 12%), Gammaproteobacteria (29% ± 6.7%), and Flavobacteriia (11.7% ± 4%), comprising approximately 73% of the microbiota (Fig. 2C). Other abundant taxa in *B. schlosseri* included Epsilonproteobacteria (7% ± 6%), Verrucomicrobia (4.7% ± 1.1%), Betaproteobacteria (1.54% ± 0.8%), Cytophagia (1.8% ± 0.4%), and Deltaproteobacteria (0.54% ± 0.34%), with diatoms comprising 7.5% ± 7.5% relative abundance (Fig. 2C; Fig. S2). Further analyses were conducted to determine microbial taxa that were enriched in *B. schlosseri* compared with surrounding seawater (Fig. 2D), revealing enrichment in *B. schlosseri* of ASVs from Alphaproteobacteria, Gammaproteobacteria, Flavobacteriia, and Epsilonproteobacteria (Fig. 2D).

Many taxa identified in our *B. schlosseri* bacterial communities are commonly found in seawater and often associated with other marine organisms. For example, Alphaproteobacteria, Gammaproteobacteria, and Flavobacteriia were previously identified in the microbiota of *B. schlosseri* and other tunicates, including *Ciona robusta*, *C. savigni*, and *B. leachi* from New Zealand (31). Alphaproteobacteria were found as dominant microbial members of *C. intestinalis* from Germany (76). Alphaproteobacteria and Gammaproteobacteria have also been shown to be highly abundant in previous studies of other tunicates (77), marine sponges (11, 78), corals (13, 79), and marine tube worms (15, 80). Bacteria from the *Rhodobacterales, Vibrionales*, and *Oceanospirillales*—belonging to the classes Alphaproteobacteria and Gammaproteobacteria, respectively, were previously isolated from *B. schlosseri* collected in the Yellow Sea, China (81). Although our results are consistent with previous findings, it remains unknown whether members of *B. schlosseri* microbial communities are permanent or transient, or if they are associated with specific tunicate body parts.

### *B. schlosseri* metabolomes are distinct from surrounding seawater

**Fig 3:**
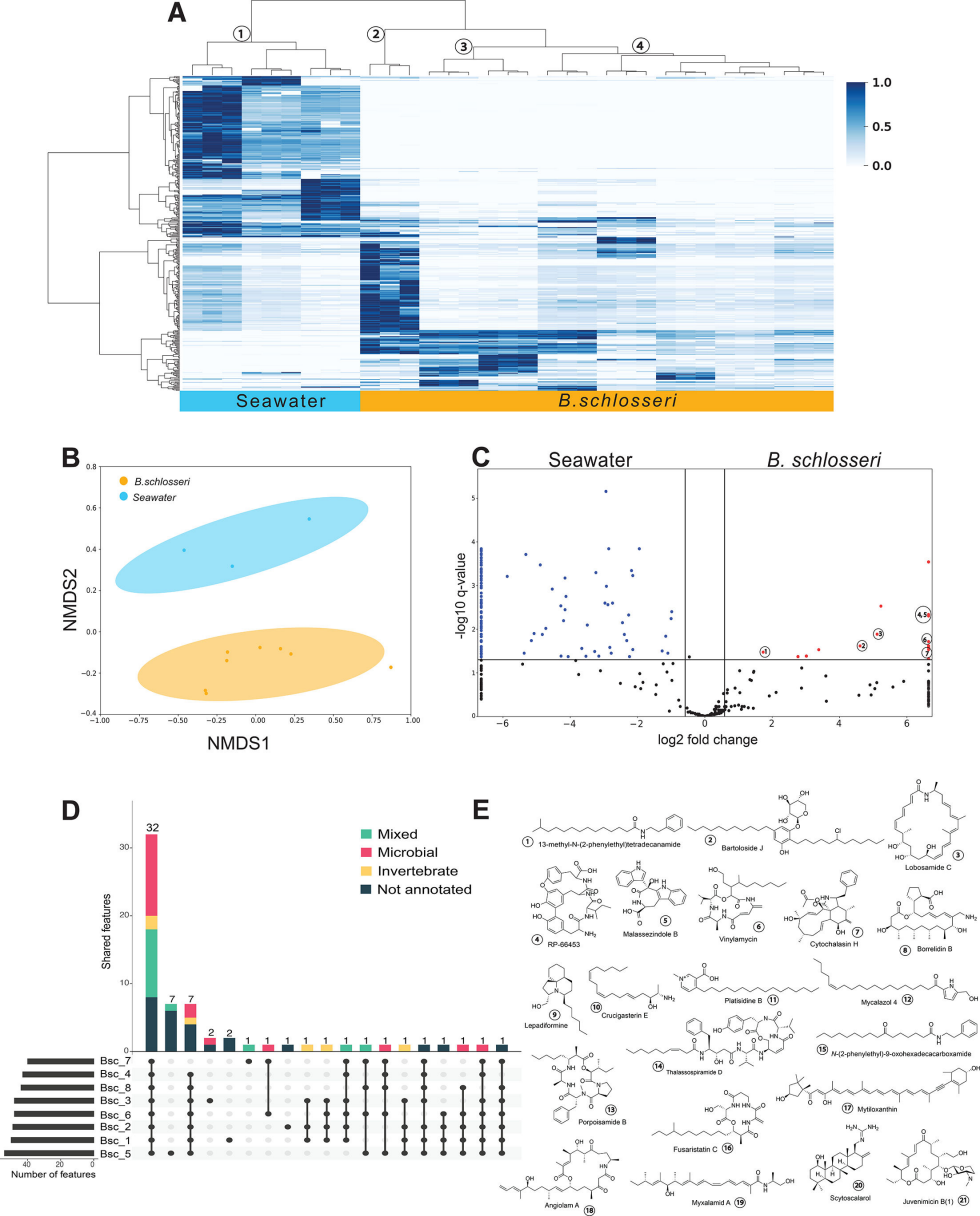
*B. schlosseri* and surrounding seawater have distinct metabolomes. (**A**) Heatmap of metabolomic features with the x-axis representing samples clustered by overall similarity using hierarchical clustering, and the y-axis representing features clustered by relative abundance across samples. Seawater and *B. schlosseri* were determined to separate into distinct subclades, with *B. schlosseri* further separating into three additional subclades. (**B**) Nonmetric multidimensional scaling (NMDS) analysis resulted in distinct clustering for *B. schlosseri* and seawater metabolomes (circles represent 95% confidence intervals; PERMANOVA *q*-value = 0.05, pseudo-*F* = 3.89). (**C**) Volcano plot comparing differential features from *B. schlosseri* and seawater resulted in prioritization of several features that were significantly more abundant in *B. schlosseri* samples. Comparison of MS1 and MS2 data resulted in putative identification of several features (**E**). (**D**) UpSet plot representing the *B. schlosseri* pan-metabolome, in which numbers above each bar indicate the total features and colors represent the proportion of annotated features, including their putative origin as reported in literature or from databases used for annotation. Metabolites of putative microbial origin are in pink, with those of putative invertebrate origin in yellow. Metabolites of mixed origin with matches to microbial and invertebrate metabolites are in green and those without annotation are dark blue. Interestingly, there are numerous features in the core metabolome (metabolites found in all samples) that are likely of microbial origin, perhaps representing compounds from consistently present microbes that may represent commensal species.

To generate metabolomes of *B. schlosseri* samples and those of surrounding seawater, we used MPACT (45) to filter and prioritize reproducible features from our MS data, which resulted in a total of 404 molecular features across all samples, with 16% of features unique to *B. schlosseri*, 35% unique to seawater, and 49% shared between both (Fig. S3). This high percentage of shared features is consistent with *B. schlosseri* as a filter feeder of the surrounding seawater. Regardless of their shared features, hierarchical clustering (Fig. 3A) revealed two distinct clades for *B. schlosseri* and seawater, suggesting distinct metabolomic profiles. Interestingly, the *B. schlosseri* clade was further divided into three subclades, with subclade 2 containing only one sample, distinct from subclades 3 and 4. This metabolomic variance among *B. schlosseri* samples, all collected at the same time and location, might suggest that microenvironment variation plays a role in the chemical ecology of *B. schlosseri*. The distinct metabolomic profiles of *B. schlosseri* and surrounding seawater were further confirmed using NMDS discriminant analysis (Fig. 3B), in which the overall metabolomic profiles were determined to form distinct groups.

To explore which features varied most between *B. schlosseri* and seawater, we constructed a volcano plot (Fig. 3C) in which we observed approximately 15 features with significantly higher abundance in *B. schlosseri* than in seawater and over 60 features with higher abundance in seawater. Thus, despite sharing almost half of their metabolomic features, significant variations in abundance of specific features may be indicative of the potential ecological roles of these metabolites in *B. schlosseri* or seawater. For example, highly abundant microbial metabolites in *B. schlosseri* may be indicative of compound sequestration or accumulation (24, 82), whereas the high diversity of metabolites in seawater may reflect the numerous chemical interactions and metabolic processes in seawater (9).

#### *B. schlosseri* metabolome composed of metabolites from diverse sources

A total of 189 features from the *B. schlosseri* and seawater metabolomes were annotated using molecular weight, formula, and/or MS fragmentation. Of the total annotated features, 56 were found only in seawater, and 39 were found only in *B. schlosseri* (Fig. S3). Of the annotated features, 78% were reported from microbial sources, including bacteria, cyanobacteria, and fungi, while 7% were reported from marine invertebrates, including octocorals, sponges, and tunicates. We categorized 15% of the annotated features as deriving from mixed source, i.e., any feature that showed matches to both microbial and invertebrate metabolites.

We then sought to further explore annotations that matched metabolites isolated from microbes. From our seawater samples, 95% of the annotated features were reported from microbial sources, such as porpoisamide (**13**) from the cyanobacteria *Lyngbya* sp. (83), thalassospiramides (**14**) from *Thalassospira* sp. (Alphaprotoebacteria) (84), and juvenimicin (**21**) from *Micromonospora chaela* (Actinobacteria) (85). In *B. schlosseri*, 46% of annotated features were from microbial sources, including from Actinobacteria, Myxobacteria*,* and Cyanobacteria. Three notable examples include RP-66453 (**4**) from *Streptomyces* spp. (86), lobosamide (**3**) from *Micromonospora* spp. (87), and myxalamid (**19**) and angiolam (**18**) from *Myxoccocus xanthus* and *Angioccocus disciformis*, respectively (88, 89). Interestingly, many of the annotated microbial metabolites from *B. schlosseri* were previously reported from microbial taxa that were found only in minor proportion in *B. schlosseri* (e.g., Deltaproteobacteria from Fig. 2, Cyanobacteria, and Actinobacteria). We did not see annotated features previously reported from *B. schlosseri*’s most abundant microbial taxa, perhaps because these metabolites have yet to be identified or perhaps reflecting bias toward metabolically rich bacteria (e.g., Actinobacteria and Cyanobacteria) in natural product databases.

We then considered annotations from our samples that matched metabolites previously isolated from invertebrates. Unfortunately, no definitive matches were found to botryllin, the only previously reported metabolite from the *Botryllus* genus. However, several annotated metabolites matched those reported from other invertebrate sources, including sponges, soft corals, and other tunicates. For example, putative annotations included the metabolites lepadiformine (**9**) and crucigasterin (**10**), which were initially isolated from the colonial tunicates *Clavelina lepadiformis* and *Pseudodistoma crucigaster*, respectively (90, 91). Another annotated invertebrate metabolite included mytiloxanthin (**17**), originally isolated from the sea mussel *Mytilus californianus* (92), but also reported from the ascidian *Halocynthia roretzi* (93). Other matches to metabolites from microbe-associated invertebrates included *N*-(phenylethyl)-9-oxohexadecacarboxamide (**15**) from the octocoral *Telesto riisei* (94) and mycalazol 4 (**12**) from the sponge *Mycale micracanthoxea* (95).

Tunicates have been recognized for their prolific production of biologically relevant metabolites (25–27, 96), although several studies have revealed that many of these interesting metabolites may be produced by associated bacteria (24, 82, 97). Our findings of both microbial- and invertebrate-derived metabolites from within our *B. schlosseri* samples are consistent with previous reports (81, 98–100). In addition, several microbial metabolites annotated from our *B. schlosseri* samples have been reported to have cytotoxic activity (101, 102), suggesting that *B. schlosseri* may have adapted to harbor associations with microorganisms capable of producing defensive compounds. Additional data related to the microbes and their metabolites consistently associated with *B. schlosseri* will allow for more definitive understanding of *B. schlosseri* microbiomes.

#### *B. schlosseri* pan-metabolome

Genomics research has been using a pan-genome approach to compare the shared and unique genomic components of related bacterial species (103, 104). More recently, the pan-genome concept has been applied to complex microbiome samples (105–107) to differentiate shared or unique microbial strains among microbiome members. We sought to apply similar concepts to our *B. schlosseri* metabolomics data, given the complexities of host-associated microbial communities, especially marine filter feeders, such as sponges and tunicates. By classifying metabolomic features in the context of a pan-metabolome, we considered the whole *B. schlosseri* metabolome as a unique entity with the host and associated microbiota (i.e., holobiont), which may form symbiotic associations contributing to each other’s defense, nutrition, protection, immunity, and/or development. Thus, we introduce the concept of a pan-metabolome to classify metabolites according to their presence or absence across complex microbiome samples. Under this premise, the core metabolome includes metabolites found in all samples, while the flexible metabolome includes those found only in some samples. Metabolites from the core metabolome would be presumed to be highly important for the biology of the system, regardless of source, whereas metabolites from the flexible metabolome might be associated with an organism’s environment or might be only transiently associated.

Among the 64 metabolomic features found in our *B. schlosseri* samples, 32 were shared across all eight samples (Fig. 3D), comprising their core metabolome. In contrast, five of the eight *B. schlosseri* samples contained features found only in that sample, one of which had seven unique features, while the other four each had either one or two unique features. The remaining three *B. schlosseri* samples shared their features with at least one other sampled colony (Fig. 3D). Those features not found in all eight samples comprise the flexible metabolome.

We were able to annotate several features in both the core and flexible metabolomes. Surprisingly, we found more features related to microbial sources in the core metabolome than in the flexible metabolome. For example, the actinomycete-derived metabolites lobosamide C (**3**, [87]) and RP-66453 (**4**, [86]) were annotated in the core metabolome. This is notable because, although members of the Actinobacteria have been reported to be tunicate-associated, they usually occur in lower abundance than members of the Proteobacteria (108). We also observed the fungal metabolite cytochalasin (**7**, [101]), produced by multiple members of the phylum Ascomycota, which has previously been reported as an important tunicate-associated phylum (24, 109).

Several features from mixed sources were annotated in the core metabolome, including sponge, coraland fungal metabolites. Crucigasterin (**10**) from the tunicate *Pseudodistoma crucigaster* (91) was found in the core metabolome, as well as fusaristatin (**16**), which has been reported from the octocoral *Eunicea fusca* and its associated fungus *Pithomyces* sp. (110). Within the flexible metabolome, seven annotated features were from microbial sources, including angiolam (**18**), scytoscalarol (**20**), and borrelidin (**8**), all discussed above. Four features were from invertebrate sources, including lepadiformine (**9**, [90]) found in seven of our *B. schlosseri* samples, as well as platisidine B (**11**, [111]), mycalazol (**12**), and *N*-(phenylethyl)-9-oxohexadecacarboxamide (**15**, [94]), all three of which were found in half of our *B. schlosseri* samples.

Additional sampling across numerous locations and various environments will be needed to confidently define members of the *B. schlosseri* pan-metabolome. However, even with our current sample size, several interesting patterns in metabolite distribution were observed, leading to the question of whether the microbial taxa producing *B. schlosseri* core metabolites might be in symbiotic association.

### Integrating the metallome, microbiome, and metabolome of *B. schlosseri* and surrounding seawater

**Fig 4:**
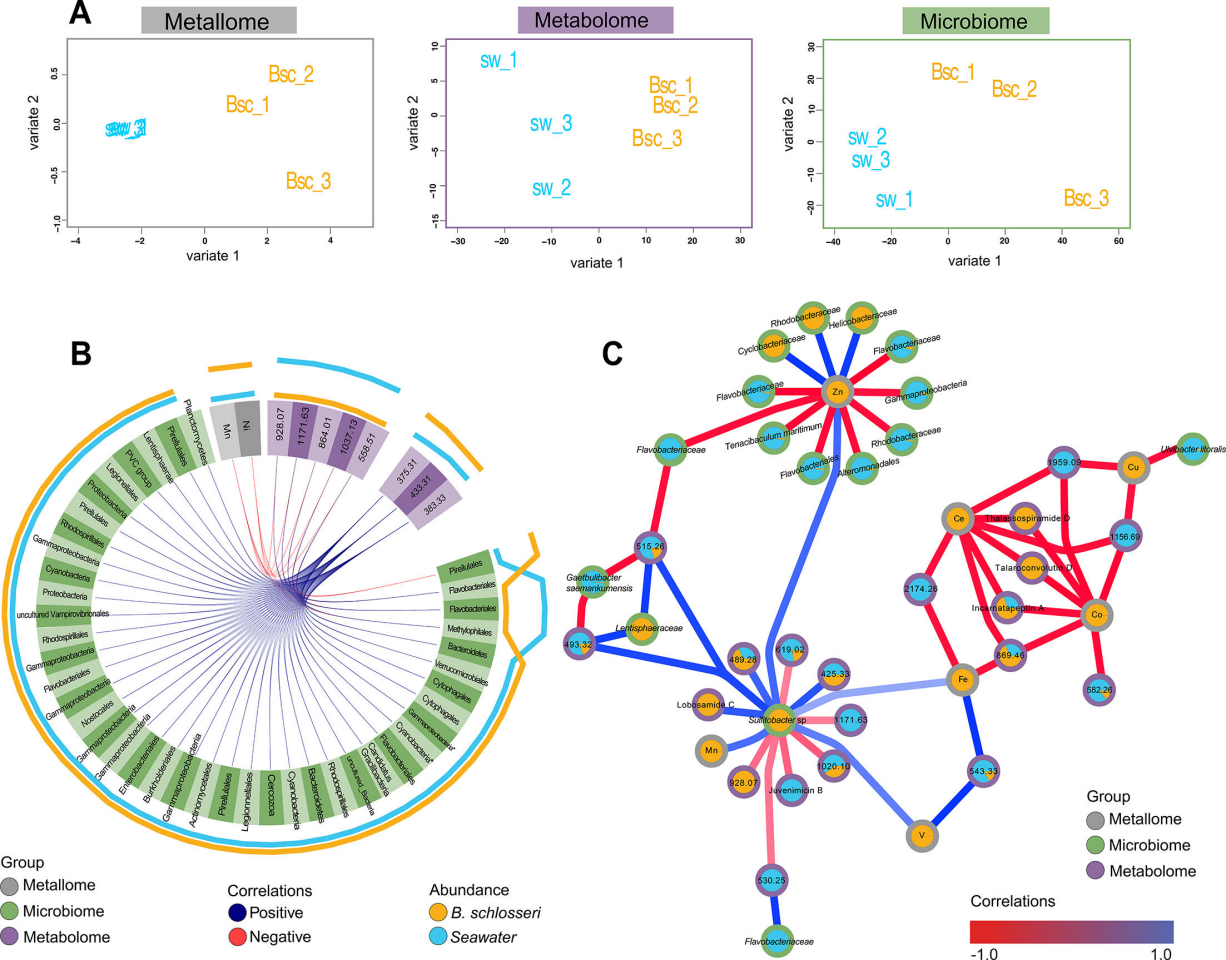
Multi-omic integration model revealed significant interactions between metals, microbes, and metabolites in *B. schlosseri* and surrounding seawater. (**A**) Individual (S) PLS-DA analyses for metallome, microbiome, and metabolome from the tuned Diablo model (M-fold validation, with 3 folds and 10 iterations) indicated that *B. schlosseri* (yellow, *n* = 3) and seawater (blue, *n* = 3) represent separate groups for each of the individual -omics analysis. (**B**) Using a circos plot built from Spearman pairwise correlations, numerous significant correlations (*r* > 0.9) were observed among members of the metallome (gray), microbiome (green), and metabolome (purple), with blue connections representing positive correlations and red connections representing negative correlations. The outer lines represent the abundance of each feature in *B. schlosseri* (yellow) and seawater (blue). (**C**) Network visualization of correlations from the DIABLO N-integration model of select multi-omics features (full network in Fig. S4). Using this integrated model, numerous intriguing relationships were apparent between specific metals, microbes, and metabolites. Node borders indicate the -omics analysis (gray for metallome, green for microbiome, purple for metabolome). Node pie charts indicate the proportion of features from *B. schlosseri* (yellow) or seawater (blue). Nodes are labeled with either the lowest microbial taxonomic resolution, metal element symbol, putative metabolite annotation, or mass in Daltons.

To further investigate how metals, microbes, and metabolites interact within *B. schlosseri* and surrounding seawater, we used an N-integration model (54) to determine multi-omics correlations. The resulting individual (s)PLS-DA analyses for metallome, microbiome, and metabolome (Fig. 4A) indicated that *B. schlosseri* and seawater represent separate groups. Interestingly, we observed tight clustering of seawater metals and moderately tight clustering of *B. schlosseri* metabolites, indicating that these groups were relatively similar for those particular -omics analyses.

Next, we sought to further integrate our multi-omics data using a circos plot to visualize both positive and negative correlations between specific features that might be driving the model (Fig. 4B). For this analysis, features with directly proportional abundances (e.g., high abundance of a specific metal correlated to high abundance of a specific metabolite or low abundance of a specific microbe correlated to low abundance of a specific metal) were denoted as positive correlations, while inverse trends in abundances between features (high versus low or low versus high) were denoted as negative correlations. Interestingly, although iron, manganese, and zinc were the most abundant metals in our *B. schlosseri* and seawater samples (Fig. 1A), in the reduced model (tuned mode) used to generate the circos plot, manganese and nickel were the metals with the most significant interactions with members of the metabolome and microbiome (Fig. 4B). The most significant correlations for manganese and nickel were all negative correlations with five unannotated metabolites, ranging from 558.5 Da to 1171.6 Da, all of which were more abundant in seawater than in *B. schlosseri*. In addition, both manganese and nickel were significantly negatively correlated with an ASV from the order *Pirellulales*; thus, as manganese and nickel increased, *Pirellulales* decreased, which might indicate sensitivity of this strain to these metals. The circos plot also revealed strong positive correlations between three unannotated features (masses of 375.3 Da, 383.3 Da, and 433.3 Da), all enriched in the *B. schlosseri* metabolome, with many microbial taxa, including members from the phyla *Proteobacteria*, *Actinobacteria*, and *Bacteroidota*.

To extend beyond pairwise correlations, we used networking analysis to visualize the N-integration model multi-omics correlations (Fig. 4C), revealing numerous interactions among metals, microbes, and metabolites. The metals cerium and cobalt were highly negatively correlated to several metabolites, including thalassopiramide D (**14**), talaroconvolutin D, and incarnatapeptin A, within *B. schlosseri* tissue. Although none of these metabolites were directly associated with iron in our study, the thalassospiramide-producing *Thalassospira* spp. are known iron-reducing bacteria (112), and talaroconvolutin D is a fungal compound that induces ferroptosis through ROS upregulation (113). Copper was also found to be negatively correlated with several metabolites, including those with masses 1959.1 and 1156.6 Da, and was also negatively correlated to *Ulvitobacter litoralis*. Copper is an important nutrient in the marine environments at trace concentrations, especially for primary producers (114); however, it has been observed that long exposure of *B. schlosseri* colonies to high concentrations of copper (e.g., 5 mg and 48 h) can be lethal (75). Additional experiments are needed to explore the role of this micronutrient in our system.

In our model (Fig. 4C), zinc exhibited negative and positive correlations with many microbial taxa, including members of Gammaproteobacteria, Flavobacteriales, and Alphaproteobacteria (Rhodobacteriales). Notably, the fish pathogen *Tenacibaculum maritimum* (Flavobacteriales), present in a much higher proportion in seawater than *B. schlosseri* tissue, was strongly negatively correlated with zinc. In contrast, a member of the family Helicobacteraceae (Epsilonproteobacteria) was positively correlated with zinc and was more abundant in *B. schlosseri* than in seawater. Previous research has shown that marine members of Helicobacteraceae are found in commensal relationships with gastropods, as well as associated with the coelomic fluid in echinoderms (115, 116), suggesting they might be beneficial host-associated bacteria, although their ecological role has not been fully described.

Iron and vanadium, both more abundant in *B. schlosseri*, were positively correlated with a member of the genus *Sulfitobacter*, which was also more abundant in *B. schlosseri* and showed further correlations with several metabolites and manganese (Fig. 4C). Iron and vanadium also exhibited positive correlations with an unannotated metabolite (mass 543.3 Da) that was more abundant in seawater. Previous research has shown bioaccumulation of iron in hemocytes from *B. schlosseri* embryos, suggesting an important role of iron in the immune system and development of *B. schlosseri* (117). Interestingly, tunicates are known to sequester vanadium to very high concentrations (118, 119), and it has been suggested that vanadium can act as a feeding deterrent for fish (120). In addition, although not well understood, there are suggestions that symbiotic bacteria might be involved in vanadium accumulation (121). For example, previous studies have found the bacterial genera *Pseudomonas* and *Ralstonia* in high abundance in vanadium-rich ascidians, particularly in the pharynx, which is involved in vanadium absorption (122). *Sulfitobacter* species are sulfite-oxidizing bacteria that have been previously shown to act as pathogens, inducing cell death in marine microalga (123), although their role in our system remains unclear, especially since both the *Sulfitobacter* sp. and essential metals, such as iron and manganese, were more abundant in *B. schlosseri*.

Many of the metals in our multi-omics integration model are essential nutrients for hosts and bacteria, including manganese, iron, cobalt, nickel, copper, and zinc, some of which have been reported to play important roles in forming pathogenic or symbiotic interactions with eukaryotic host cells (124). For example, in mammalian cells, the immune system inhibits bacterial growth by withholding critical metals—including manganese, iron, and zinc—to starve invading bacteria, a process known as nutritional immunity (125, 126). In other eukaryotic hosts, there is also evidence of increased concentrations of copper, zinc, iron, and manganese as part of host defenses against bacteria, especially during colonization (127–129). Additional experiments are needed to further explore the ecological roles that associated bacterial taxa and metabolites in the context of *B. schlosseri* metal binding, accumulation, and transport.

### Conclusion

Herein, we compared the microbes, metals, and metabolites found in *B. schlosseri* with those in the surrounding seawater. We identified taxa within the microbial community of *B. schlosseri* that were either more abundant than in seawater (e.g., *Gammaproteobacteria* and *Epsilonproteobacteria*) or less abundant (e.g., *Alphaproteobacteria* and *Flavobacteriia*), suggesting that *B. schlosseri* employs some mechanism(s) for microbiome selection. Similarly, our baseline metallome studies demonstrated that *B. schlosseri* sequesters several metals with substantial fold increases over those found in seawater. We also found several microbial metabolites to be more abundant in *B. schlosseri* than in seawater, including some that may bind metals based on their chemical structures. We introduced the concept of a pan-metabolome to classify metabolites of high importance based on their presence or absence across samples, finding that *B. schlosseri* samples had a high proportion of shared metabolites, indicating a closed pan-metabolome. Finally, we integrated our multi-omics data to investigate which microbial taxa and metabolites co-vary with metals, identifying several that are positively or negatively correlated. These results suggest that metals and metabolites play important roles in structuring the *B. schlosseri* microbiome, making it an ideal test system for future mechanistic investigation and for unraveling possible symbiotic relationships based on metabolite interactions with microbes and metals.

This study is the first multi-omics approach connecting the metallome to the microbiome and metabolome of *B. schlosseri*. Tunicates, including *B. schlosseri*, are integral members of the marine environment, providing important, bioactive natural products, with recent interest in the role of their associated microbes. However, while some tunicate taxa have been studied for their ability to bioaccumulate trace metals (29, 30), the microbial role in this process—as well as that of associated metabolites—remains poorly understood. Our study bridges the knowledge gap surrounding the intricate relationships among organisms, their microbiomes, the metabolites they produce, and the sequestered and environmental metals. The correlations we observed reveal the important interactions between metals, microbes, and metabolites in *B. schlosseri*, providing a baseline for future studies of metal accumulation from a host-microbiome context. Moreover, this research establishes a foundation for comprehending the ecological importance of trace metals in host-microbe systems, potentially paving the way for the discovery of new natural products with unique therapeutic applications.

## Data Availability

Sequencing data were deposited to the National Center for Biotechnology Information (NCBI) GenBank (https://www.ncbi.nlm.nih.gov/genbank/) under accession number PRJNA1039848. Metabolomics data were deposited to the Mass Spectrometry Interactive Virtual Environment repository (MassIVE; https://massive.ucsd.edu/ProteoSAFe/dataset.jsp?task=6b6b031e177043cfbbbc5546bf0ccc8d) under accession number MSV000096313. Code is available on GitHub (https://github.com/BalunasLab/Botryllus-schlosseri multiomics).

## SUPPLEMENTAL MATERIAL

Supplemental material msystems.00793-25-s0001.docx Supplemental figures and tables.

ASM does not own the copyrights to Supplemental Material that may be linked to, or accessed through, an article. The authors have granted ASM a non-exclusive, world-wide license to publish the Supplemental Material files. Please contact the corresponding author directly for reuse.

## References

[1] Bellante A, Piazzese D, Cataldo S, Parisi MG, Cammarata M. 2016. Evaluation and comparison of trace metal accumulation in different tissues of potential bioindicator organisms: macrobenthic filter feeders Styela plicata, Sabella spallanzanii, and Mytilus galloprovincialis*.* Environ Toxicol Chem 35:3062–3070. doi:10.1002/etc.3494 27187528

[2] Lane TW, Morel FMM. 2000. A biological function for cadmium in marine diatoms. Proc Natl Acad Sci USA 97:4627–4631. doi:10.1073/pnas.090091397 10781068 PMC18283

[3] Morel FMM, Price NM. 2003. The biogeochemical cycles of trace metals in the oceans. Science 300:944–947. doi:10.1126/science.1083545 12738853

[4] Martin JH, Knauer GA. 1973. The elemental composition of plankton. Geochim Cosmochim Acta 37:1639–1653. doi:10.1016/0016-7037(73)90154-3

[5] Boyd RS. 2010. Heavy metal pollutants and chemical ecology: exploring new frontiers. J Chem Ecol 36:46–58. doi:10.1007/s10886-009-9730-5 20108028

[6] Boyd PW, Ellwood MJ. 2010. The biogeochemical cycle of iron in the ocean. Nature Geosci 3:675–682. doi:10.1038/ngeo964

[7] Sánchez-Quiles D, Marbà N, Tovar-Sánchez A. 2017. Trace metal accumulation in marine macrophytes: hotspots of coastal contamination worldwide. Sci Total Environ 576:520–527. doi:10.1016/j.scitotenv.2016.10.144 27810741

[8] Tripathi P, Singhal A, Jha PK. 2022. Metal transport and its impact on coastal ecosystem, p 239–264. In Madhav S, Nazneen S, Singh P (ed), Coastal ecosystems: environmental importance, current challenges and conservation measures. Springer International Publishing, Cham.

[9] Stocker R. 2012. Marine microbes see a sea of gradients. Science 338:628–633. doi:10.1126/science.1208929 23118182

[10] Flemming H-C, Wuertz S. 2019. Bacteria and archaea on earth and their abundance in biofilms. Nat Rev Microbiol 17:247–260. doi:10.1038/s41579-019-0158-9 30760902

[11] Pita L, Rix L, Slaby BM, Franke A, Hentschel U. 2018. The sponge holobiont in a changing ocean: from microbes to ecosystems. Microbiome 6:46. doi:10.1186/s40168-018-0428-1 29523192 PMC5845141

[12] O’Brien PA, Webster NS, Miller DJ, Bourne DG. 2019. Host-microbe coevolution: applying evidence from model systems to complex marine invertebrate holobionts. mBio 10:e02241-18. doi:10.1128/mBio.02241-18 30723123 PMC6428750

[13] Bourne DG, Dennis PG, Uthicke S, Soo RM, Tyson GW, Webster N. 2013. Coral reef invertebrate microbiomes correlate with the presence of photosymbionts. ISME J 7:1452–1458. doi:10.1038/ismej.2012.172 23303372 PMC3695284

[14] Stewart FJ, Cavanaugh CM. 2006. Symbiosis of thioautotrophic bacteria with Riftia pachyptila. Prog Mol Subcell Biol 41:197–225. doi:10.1007/3-540-28221-1_10 16623395

[15] Vijayan N, Lema KA, Nedved BT, Hadfield MG. 2019. Microbiomes of the polychaete Hydroides elegans (Polychaeta: Serpulidae) across its life-history stages. Mar Biol 166:19. doi:10.1007/s00227-019-3465-9

[16] McFall-Ngai M, Hadfield MG, Bosch TCG, Carey HV, Domazet-Lošo T, Douglas AE, Dubilier N, Eberl G, Fukami T, Gilbert SF, et al.. 2013. Animals in a bacterial world, a new imperative for the life sciences. Proc Natl Acad Sci USA 110:3229–3236. doi:10.1073/pnas.1218525110 23391737 PMC3587249

[17] Unzueta-Martínez A, Welch H, Bowen JL. 2021. Determining the composition of resident and transient members of the oyster microbiome. Front Microbiol 12:828692. doi:10.3389/fmicb.2021.828692 35185836 PMC8847785

[18] Kerwin AH, Gromek SM, Suria AM, Samples RM, Deoss DJ, O’Donnell K, Frasca S Jr, Sutton DA, Wiederhold NP, Balunas MJ, Nyholm SV. 2019. Shielding the next generation: symbiotic bacteria from a reproductive organ protect bobtail squid eggs from fungal fouling. mBio 10:e02376-19. doi:10.1128/mBio.02376-19 PMC6819662 31662458

[19] Maansson M, Vynne NG, Klitgaard A, Nybo JL, Melchiorsen J, Nguyen DD, Sanchez LM, Ziemert N, Dorrestein PC, Andersen MR, Gram L. 2016. An integrated metabolomic and genomic mining workflow to uncover the biosynthetic potential of bacteria. mSystems 1:e00028-15. doi:10.1128/mSystems.00028-15 PMC5069768 27822535

[20] Mannochio-Russo H, Swift SOI, Nakayama KK, Wall CB, Gentry EC, Panitchpakdi M, Caraballo-Rodriguez AM, Aron AT, Petras D, Dorrestein K, et al.. 2023. Microbiomes and metabolomes of dominant coral reef primary producers illustrate a potential role for immunolipids in marine symbioses. Commun Biol 6:896. doi:10.1038/s42003-023-05230-1 37653089 PMC10471604

[21] Erwin PM, Pineda MC, Webster N, Turon X, López-Legentil S. 2014. Down under the tunic: bacterial biodiversity hotspots and widespread ammonia-oxidizing archaea in coral reef ascidians. ISME J 8:575–588. doi:10.1038/ismej.2013.188 24152714 PMC3930322

[22] Menezes CBA, Bonugli-Santos RC, Miqueletto PB, Passarini MRZ, Silva CHD, Justo MR, Leal RR, Fantinatti-Garboggini F, Oliveira VM, Berlinck RGS, Sette LD. 2010. Microbial diversity associated with algae, ascidians and sponges from the north coast of São Paulo state, Brazil. Microbiol Res 165:466–482. doi:10.1016/j.micres.2009.09.005 19879115

[23] Donia MS, Fricke WF, Partensky F, Cox J, Elshahawi SI, White JR, Phillippy AM, Schatz MC, Piel J, Haygood MG, Ravel J, Schmidt EW. 2011. Complex microbiome underlying secondary and primary metabolism in the tunicate-prochloron symbiosis. Proc Natl Acad Sci USA 108:E1423–E1432. doi:10.1073/pnas.1111712108 22123943 PMC3251135

[24] Bauermeister A, Branco PC, Furtado LC, Jimenez PC, Costa-Lotufo LV, da Cruz Lotufo TM. 2018. Tunicates: a model organism to investigate the effects of associated-microbiota on the production of pharmaceuticals. Drug Discovery Today: Disease Models 28:13–20. doi:10.1016/j.ddmod.2019.08.008

[25] Raub MF, Cardellina JH, Choudhary MI, Ni CZ, Clardy J, Alley MC. 1991. Clavepictines A and B: cytotoxic quinolizidines from the tunicate Clavelina picta. J Am Chem Soc 113:3178–3180. doi:10.1021/ja00008a060

[26] D’Incalci M, Galmarini CM. 2010. A review of trabectedin (ET-743): a unique mechanism of action. Mol Cancer Ther 9:2157–2163. doi:10.1158/1535-7163.MCT-10-0263 20647340

[27] Nuzzo G, Gallo C, Crocetta F, Romano L, Barra G, Senese G, dell’Isola M, Carbone D, Tanduo V, Albiani F, Villani G, d’Ippolito G, Manzo E, Fontana A. 2022. Identification of the marine alkaloid lepadin A as potential inducer of immunogenic cell death. Biomolecules 12:246. doi:10.3390/biom12020246 35204747 PMC8961536

[28] Ramesh C, Tulasi BR, Raju M, Thakur N, Dufossé L. 2021. Marine natural products from tunicates and their associated microbes. Mar Drugs 19:308. doi:10.3390/md19060308 34073515 PMC8228501

[29] Radhalakshmi R, Sivakumar V, Ali HAJ. 2014. Analysis of selected species of ascidians as bioindicators of metals in marine ecosystem. Int J Curr Microbiol App Sci 3:755–764.

[30] Tzafriri-Milo R, Benaltabet T, Torfstein A, Shenkar N. 2019. The potential use of invasive ascidians for biomonitoring heavy metal pollution. Front Mar Sci 6:611. doi:10.3389/fmars.2019.00611

[31] Cahill PL, Fidler AE, Hopkins GA, Wood SA. 2016. Geographically conserved microbiomes of four temperate water tunicates. Environ Microbiol Rep 8:470–478. doi:10.1111/1758-2229.12391 26929150

[32] Goldstein O, Mandujano-Tinoco EA, Levy T, Talice S, Raveh T, Gershoni-Yahalom O, Voskoboynik A, Rosental B. 2021. Botryllus schlosseri as a unique colonial chordate model for the study and modulation of innate immune activity. Mar Drugs 19:1–12. doi:10.3390/md19080454 PMC8398012 34436293

[33] Rosental B, Raveh T, Voskoboynik A, Weissman IL. 2020. Evolutionary perspective on the hematopoietic system through a colonial chordate: allogeneic immunity and hematopoiesis. Curr Opin Immunol 62:91–98. doi:10.1016/j.coi.2019.12.006 31954962 PMC7136747

[34] Manni L, Anselmi C, Cima F, Gasparini F, Voskoboynik A, Martini M, Peronato A, Burighel P, Zaniolo G, Ballarin L. 2019. Sixty years of experimental studies on the blastogenesis of the colonial tunicate Botryllus schlosseri. Dev Biol 448:293–308. doi:10.1016/j.ydbio.2018.09.009 30217596

[35] Taketa DA, De Tomaso AW. 2015. Botryllus schlosseri allorecognition: tackling the enigma. Dev Comp Immunol 48:254–265. doi:10.1016/j.dci.2014.03.014 24709050 PMC4185259

[36] Schreiber L, Kjeldsen KU, Funch P, Jensen J, Obst M, López-Legentil S, Schramm A. 2016. Endozoicomonas are specific, facultative symbionts of sea squirts. Front Microbiol 7:1042. doi:10.3389/fmicb.2016.01042 27462299 PMC4940369

[37] McDaniel WH. 1996. Method 200.3 sample preparation procedure for spectrochemical determination of total recoverable elements in biological tissues

[38] US EPA. 1998. Method 6020A (SW-846): inductively coupled plasma-mass spectrometry. Cincinnati

[39] US EPA. 2019. Method 200.7: determination of metals and trace elements in water and wastes by inductively coupled plasma-atomic emission spectrometry. Available from: https://www.epa.gov/esam/method-2007-determination-metals-and-trace-elements-water-and-wastes-inductively-coupled. Retrieved 06 Nov 2024.

[40] Bolyen E, Rideout JR, Dillon MR, Bokulich NA, Abnet CC, Al-Ghalith GA, Alexander H, Alm EJ, Arumugam M, Asnicar F, et al.. 2019. Reproducible, interactive, scalable and extensible microbiome data science using QIIME 2. Nat Biotechnol 37:852–857. doi:10.1038/s41587-019-0209-9 31341288 PMC7015180

[41] Callahan BJ, McMurdie PJ, Rosen MJ, Han AW, Johnson AJA, Holmes SP. 2016. DADA2: high-resolution sample inference from Illumina amplicon data. Nat Methods 13:581–583. doi:10.1038/nmeth.3869 27214047 PMC4927377

[42] DeSantis TZ, Hugenholtz P, Larsen N, Rojas M, Brodie EL, Keller K, Huber T, Dalevi D, Hu P, Andersen GL. 2006. Greengenes, a chimera-checked 16S rRNA gene database and workbench compatible with ARB. Appl Environ Microbiol 72:5069–5072. doi:10.1128/AEM.03006-05 16820507 PMC1489311

[43] Bray JR, Curtis JT. 1957. An ordination of the upland forest communities of southern Wisconsin. Ecol Monogr 27:325–349. doi:10.2307/1942268

[44] Chen J, Bittinger K, Charlson ES, Hoffmann C, Lewis J, Wu GD, Collman RG, Bushman FD, Li H. 2012. Associating microbiome composition with environmental covariates using generalized UniFrac distances. Bioinformatics 28:2106–2113. doi:10.1093/bioinformatics/bts342 22711789 PMC3413390

[45] Samples RM, Puckett SP, Balunas MJ. 2023. Metabolomics peak analysis computational tool (MPACT): an advanced informatics tool for metabolomics and data visualization of molecules from complex biological samples. Anal Chem 95:8770–8779. doi:10.1021/acs.analchem.2c04632 37260127

[46] Dührkop K, Fleischauer M, Ludwig M, Aksenov AA, Melnik AV, Meusel M, Dorrestein PC, Rousu J, Böcker S. 2019. SIRIUS 4: a rapid tool for turning tandem mass spectra into metabolite structure information. Nat Methods 16:299–302. doi:10.1038/s41592-019-0344-8 30886413

[47] van Santen JA, Jacob G, Singh AL, Aniebok V, Balunas MJ, Bunsko D, Neto FC, Castaño-Espriu L, Chang C, Clark TN, et al.. 2019. The natural products atlas: an open access knowledge base for microbial natural products discovery. ACS Cent Sci 5:1824–1833. doi:10.1021/acscentsci.9b00806 31807684 PMC6891855

[48] MarinLit. 2024. A database of the marine natural products literature*.* Available from: https://marinlit.rsc.org/

[49] Allen F, Pon A, Wilson M, Greiner R, Wishart D. 2014. CFM-ID: a web server for annotation, spectrum prediction and metabolite identification from tandem mass spectra. Nucleic Acids Res 42:W94–W99. doi:10.1093/nar/gku436 24895432 PMC4086103

[50] Schymanski EL, Jeon J, Gulde R, Fenner K, Ruff M, Singer HP, Hollender J. 2014. Identifying small molecules via high resolution mass spectrometry: communicating confidence. Environ Sci Technol 48:2097–2098. doi:10.1021/es5002105 24476540

[51] Benjamini Y, Hochberg Y. 1995. Controlling the false discovery rate: a practical and powerful approach to multiple testing. J R Stat Soc B 57:289–300. doi:10.1111/j.2517-6161.1995.tb02031.x

[52] Voorhees EM. The effectiveness and efficiency of agglomerative hierarchic clustering in document retrieval PhD thesis, Cornell University, New York

[53] Conway JR, Lex A, Gehlenborg N. 2017. UpSetR: an R package for the visualization of intersecting sets and their properties. Bioinformatics 33:2938–2940. doi:10.1093/bioinformatics/btx364 28645171 PMC5870712

[54] Singh A, Shannon CP, Gautier B, Rohart F, Vacher M, Tebbutt SJ, Lê Cao K-A. 2019. DIABLO: an integrative approach for identifying key molecular drivers from multi-omics assays. Bioinformatics 35:3055–3062. doi:10.1093/bioinformatics/bty1054 30657866 PMC6735831

[55] Rohart F, Gautier B, Singh A, Lê Cao K-A. 2017. mixOmics: An R package for ’omics feature selection and multiple data integration. PLoS Comput Biol 13:e1005752. doi:10.1371/journal.pcbi.1005752 29099853 PMC5687754

[56] van den Berg RA, Hoefsloot HCJ, Westerhuis JA, Smilde AK, van der Werf MJ. 2006. Centering, scaling, and transformations: improving the biological information content of metabolomics data. BMC Genomics 7:142. doi:10.1186/1471-2164-7-142 16762068 PMC1534033

[57] Nearing JT, Douglas GM, Hayes MG, MacDonald J, Desai DK, Allward N, Jones CMA, Wright RJ, Dhanani AS, Comeau AM, Langille MGI. 2022. Microbiome differential abundance methods produce different results across 38 datasets. Nat Commun 13:342. doi:10.1038/s41467-022-28034-z 35039521 PMC8763921

[58] Strayer N, Zhang S, Yao L, Vessels T, Bejan CA, Hsi RS, Shirey-Rice JK, Balko JM, Johnson DB, Phillips EJ, Bick A, Edwards TL, Velez Edwards DR, Pulley JM, Wells QS, Savona MR, Cox NJ, Roden DM, Ruderfer DM, Xu Y. 2023. Interactive network-based clustering and investigation of multimorbidity association matrices with association subgraphs. Bioinformatics 39:1–6. doi:10.1093/bioinformatics/btac780 PMC9825768 36472455

[59] Shannon P, Markiel A, Ozier O, Baliga NS, Wang JT, Ramage D, Amin N, Schwikowski B, Ideker T. 2003. Cytoscape: a software environment for integrated models of biomolecular interaction networks. Genome Res 13:2498–2504. doi:10.1101/gr.1239303 14597658 PMC403769

[60] Chester R, Stoner JH. 1974. The distribution of zinc, nickel, manganese, cadmium, copper, and iron in some surface waters from the world ocean. Mar Chem 2:17–32. doi:10.1016/0304-4203(74)90003-6

[61] Lagerström ME, Field MP, Séguret M, Fischer L, Hann S, Sherrell RM. 2013. Automated on-line flow-injection ICP-MS determination of trace metals (Mn, Fe, Co, Ni, Cu and Zn) in open ocean seawater: application to the GEOTRACES program. Mar Chem 155:71–80. doi:10.1016/j.marchem.2013.06.001

[62] Wysocka I, Vassileva E. 2017. Method validation for high resolution sector field inductively coupled plasma mass spectrometry determination of the emerging contaminants in the open ocean: rare earth elements as a case study. Spectrochim Acta B 128:1–10. doi:10.1016/j.sab.2016.12.004

[63] Samanta S, Cloete R, Loock J, Rossouw R, Roychoudhury AN. 2021. Determination of trace metal (Mn, Fe, Ni, Cu, Zn, Co, Cd and Pb) concentrations in seawater using single quadrupole ICP-MS: a comparison between offline and online preconcentration setups. Minerals 11:1289. doi:10.3390/min11111289

[64] Zhao L, Xia Z, Wang F. 2014. Zebrafish in the sea of mineral (iron, zinc, and copper) metabolism. Front Pharmacol 5:33. doi:10.3389/fphar.2014.00033 24639652 PMC3944790

[65] Donaghy C, Javellana JG, Hong Y-J, Djoko K, Angeles-Boza AM. 2023. The synergy between zinc and antimicrobial peptides: an insight into unique bioinorganic interactions. Molecules 28:2156. doi:10.3390/molecules28052156 36903402 PMC10004757

[66] Hara T, Takeda T-A, Takagishi T, Fukue K, Kambe T, Fukada T. 2017. Physiological roles of zinc transporters: molecular and genetic importance in zinc homeostasis. J Physiol Sci 67:283–301. doi:10.1007/s12576-017-0521-4 28130681 PMC10717645

[67] Skrajnowska D, Bobrowska-Korczak B. 2019. Role of zinc in immune system and anti-cancer defense mechanisms. Nutrients 11:2273. doi:10.3390/nu11102273 31546724 PMC6835436

[68] Holley AK, Bakthavatchalu V, Velez-Roman JM, St Clair DK. 2011. Manganese superoxide dismutase: guardian of the powerhouse. Int J Mol Sci 12:7114–7162. doi:10.3390/ijms12107114 22072939 PMC3211030

[69] Li L, Yang X. 2018. The essential element manganese, oxidative stress, and metabolic diseases: links and interactions. Oxid Med Cell Longev 2018:7580707. doi:10.1155/2018/7580707 29849912 PMC5907490

[70] Abbaspour N, Hurrell R, Kelishadi R. 2014. Review on iron and its importance for human health. J Res Med Sci 19:164–174. 24778671 PMC3999603

[71] Hogle SL, Barbeau KA, Gledhill M. 2014. Heme in the marine environment: from cells to the iron cycle. Metallomics 6:1107–1120. doi:10.1039/c4mt00031e 24811388

[72] Strohal P, Tuta J, Kolar Z. 1969. Investigations of certain microconstituents in two tunicates1. Limnol Oceanogr 14:265–268. doi:10.4319/lo.1969.14.2.0265

[73] Huang J, Yu Z, Chistoserdova L. 2018. Lanthanide-dependent methanol dehydrogenases of XoxF4 and XoxF5 clades are differentially distributed among methylotrophic bacteria and they reveal different biochemical properties. Front Microbiol 9:1366. doi:10.3389/fmicb.2018.01366 29997591 PMC6028718

[74] Xu C, Qu X. 2014. Cerium oxide nanoparticle: a remarkably versatile rare earth nanomaterial for biological applications. NPG Asia Mater 6:e90–e90. doi:10.1038/am.2013.88

[75] Gregorin C, Albarano L, Somma E, Costantini M, Zupo V. 2021. Assessing the ecotoxicity of copper and polycyclic aromatic hydrocarbons: comparison of effects on Paracentrotus lividus and Botryllus schlosseri, as alternative bioassay methods. Water (Basel) 13:711. doi:10.3390/w13050711

[76] Dishaw LJ, Flores-Torres J, Lax S, Gemayel K, Leigh B, Melillo D, Mueller MG, Natale L, Zucchetti I, De Santis R, Pinto MR, Litman GW, Gilbert JA. 2014. The gut of geographically disparate Ciona intestinalis harbors a core microbiota. PLoS One 9:e93386. doi:10.1371/journal.pone.0093386 24695540 PMC3973685

[77] Wei J, Gao H, Yang Y, Liu H, Yu H, Chen Z, Dong B. 2020. Seasonal dynamics and starvation impact on the gut microbiome of urochordate ascidian Halocynthia roretzi. Anim Microbiome 2:30. doi:10.1186/s42523-020-00048-2 33499981 PMC7807810

[78] Webster NS, Taylor MW. 2012. Marine sponges and their microbial symbionts: love and other relationships. Environ Microbiol 14:335–346. doi:10.1111/j.1462-2920.2011.02460.x 21443739

[79] Jensen S, Hovland M, Lynch MDJ, Bourne DG. 2019. Diversity of deep-water coral-associated bacteria and comparison across depth gradients. FEMS Microbiol Ecol 95:fiz091. doi:10.1093/femsec/fiz091 31210258

[80] Fuirst M, Ward CS, Schwaner C, Diana Z, Schultz TF, Rittschof D. 2021. Compositional and functional microbiome variation between tubes of an intertidal polychaete and surrounding marine sediment. Front Mar Sci 8:656506. doi:10.3389/fmars.2021.656506

[81] Chen L, Liu C, Liu X, Wang G-Y. 2020. Phylogenetic analysis and screening of antimicrobial and cytotoxic activities of culturable bacteria associated with the ascidian Botryllus schlosseri. J Appl Microbiol 129:892–905. doi:10.1111/jam.14667 32311814

[82] Schmidt EW. 2015. The secret to a successful relationship: lasting chemistry between ascidians and their symbiotic bacteria. Invertebr Biol 134:88–102. doi:10.1111/ivb.12071 25937788 PMC4414342

[83] Meickle T, Gunasekera SP, Liu Y, Luesch H, Paul VJ. 2011. Porpoisamides A and B, two novel epimeric cyclic depsipeptides from a Florida keys collection of Lyngbya sp. Bioorg Med Chem 19:6576–6580. doi:10.1016/j.bmc.2011.05.051 21705224 PMC3197893

[84] Oh DC, Strangman WK, Kauffman CA, Jensen PR, Fenical W. 2007. Thalassospiramides A and B, immunosuppressive peptides from the marine bacterium Thalassospira sp. Org Lett 9:1525–1528. doi:10.1021/ol070294u 17373804

[85] Kishi T, Harada S, Yamana H, Miyake A. 1976. Studies on juvenimicin, a new antibiotic. II. Isolation, chemical characterization and structures. J Antibiot 29:1171–1181. doi:10.7164/antibiotics.29.1171 993104

[86] Helynck G, Dubertret C, Frechet D, Leboul J. 1998. Isolation of RP 66453, a new secondary peptide metabolite from Streptomyces sp. useful as a lead for neurotensin antagonists. J Antibiot 51:512–514. doi:10.7164/antibiotics.51.512 9666181

[87] Schulze CJ, Donia MS, Siqueira-Neto JL, Ray D, Raskatov JA, Green RE, McKerrow JH, Fischbach MA, Linington RG. 2015. Genome-directed lead discovery: biosynthesis, structure elucidation, and biological evaluation of two families of polyene macrolactams against Trypanosoma brucei*.* ACS Chem Biol 10:2373–2381. doi:10.1021/acschembio.5b00308 26270237 PMC7505085

[88] Gerth K, Jansen R, Reifenstahl G, Höfle G, Irschik H, Kunze B, Reichenbach H, Thierbach G. 1983. The myxalamids, new antibiotics from Myxococcus xanthus (Myxobacterales) I. production, physico-chemical and biological properties, and mechanism of action. J Antibiot 36:1150–1156. doi:10.7164/antibiotics.36.1150 6415031

[89] Kunze B, Kohl W, Hofle G, Reichenbach H. 1985. Production, isolation, physico-chemical and biological properties of angiolam A, a new antibiotic from Angiococcus disciformis (Myxobacterales). J Antibiot 38:1649–1654. doi:10.7164/antibiotics.38.1649 3937837

[90] Biard JF, Guyot S, Roussakis C, Verbist JF, Vercauteren J, Weber JF, Boukef K. 1994. Lepadiformine, a new marine cytotoxic alkaloid from Clavelina lepadiformis müller. Tetrahedron Lett 35:2691–2694. doi:10.1016/S0040-4039(00)77007-9

[91] Ciavatta ML, Manzo E, Nuzzo G, Villani G, Varcamonti M, Gavagnin M. 2010. Crucigasterins A–E, antimicrobial amino alcohols from the mediterranean colonial ascidian Pseudodistoma crucigaster*.* Tetrahedron 66:7533–7538. doi:10.1016/j.tet.2010.07.056

[92] Scheer BT. 1940. Some features of the metabolism of the carotenoid pigments in the California sea mussel (Mytilus californianus). J Biol Chem 136:275–299. doi:10.1016/S0021-9258(18)73099-8

[93] Ookubo M, Matsuno T. 1985. Carotenoids of sea squirts—II. Comparative biochemical studies of carotenoids in sea squirts. Comp Biochem Physiol B Comp Biochem 81:137–141. doi:10.1016/0305-0491(85)90174-9

[94] Liyanage GK, Schmitz FJ. 1996. Cytotoxic amides from the octocoral Telesto riisei*.* J Nat Prod 59:148–151. doi:10.1021/np960032t 8991947

[95] Ortega MJ, Zubia E, Luis Carballo J, Salvá J. 1997. New cytotoxic metabolites from the sponge Mycale micracanthoxea. Tetrahedron 53:331–340. doi:10.1016/S0040-4020(96)00989-1

[96] Palanisamy SK, Rajendran NM, Marino A. 2017. Natural products diversity of marine ascidians (tunicates; ascidiacea) and successful drugs in clinical development. Nat Prod Bioprospect 7:1–111. doi:10.1007/s13659-016-0115-5 28097641 PMC5315671

[97] Ayuningrum D, Liu Y, Sibero MT, Kristiana R, Asagabaldan MA, Wuisan ZG, Trianto A, Radjasa OK, Sabdono A, Schäberle TF. 2019. Tunicate-associated bacteria show a great potential for the discovery of antimicrobial compounds. PLoS One 14:e0213797. doi:10.1371/journal.pone.0213797 30875400 PMC6420000

[98] Usov AI, Slanchev KI, Smirnova GP, Ivanova AP, Stefanov KL, Popov SS, Andreev StN. 2002. Polar constituents of the tunicate Botryllus schlosseri. Russ J Bioorganic Chem 28:147–151. doi:10.1023/A:1015073525159 11962238

[99] Menin A, Ballarin L. 2008. Immunomodulatory molecules in the compound ascidian Botryllus schlosseri: evidence from conditioned media. J Invertebr Pathol 99:275–280. doi:10.1016/j.jip.2008.08.001 18768139

[100] Franchi N, Ballarin L, Cima F. 2023. Botryllin, a novel antimicrobial peptide from the colonial ascidian Botryllus schlosseri. Mar Drugs 21:74. doi:10.3390/md21020074 36827115 PMC9966394

[101] Lee J, Yi J-M, Kim H, Lee YJ, Park J-S, Bang O-S, Kim NS. 2014. Cytochalasin H, an active anti-angiogenic constituent of the ethanol extract of Gleditsia sinensis thorns. Biol Pharm Bull 37:6–12. doi:10.1248/bpb.b13-00318 24172060

[102] Schulze CJ, Bray WM, Loganzo F, Lam M-H, Szal T, Villalobos A, Koehn FE, Linington RG. 2014. Borrelidin B: isolation, biological activity, and implications for nitrile biosynthesis. J Nat Prod 77:2570–2574. doi:10.1021/np500727g 25393949

[103] Brockhurst MA, Harrison E, Hall JPJ, Richards T, McNally A, MacLean C. 2019. The ecology and evolution of pangenomes. Curr Biol 29:R1094–R1103. doi:10.1016/j.cub.2019.08.012 31639358

[104] Golicz AA, Bayer PE, Bhalla PL, Batley J, Edwards D. 2020. Pangenomics comes of age: from bacteria to plant and animal applications. Trends Genet 36:132–145. doi:10.1016/j.tig.2019.11.006 31882191

[105] Delmont TO, Eren AM. 2018. Linking pangenomes and metagenomes: the Prochlorococcus metapangenome. PeerJ 6:e4320. doi:10.7717/peerj.4320 29423345 PMC5804319

[106] Utter DR, Borisy GG, Eren AM, Cavanaugh CM, Mark Welch JL. 2020. Metapangenomics of the oral microbiome provides insights into habitat adaptation and cultivar diversity. Genome Biol 21:293. doi:10.1186/s13059-020-02200-2 33323129 PMC7739467

[107] Zhong C, Chen C, Wang L, Ning K. 2021. Integrating pan-genome with metagenome for microbial community profiling. Comput Struct Biotechnol J 19:1458–1466. doi:10.1016/j.csbj.2021.02.021 33841754 PMC8010324

[108] Tianero MDB, Kwan JC, Wyche TP, Presson AP, Koch M, Barrows LR, Bugni TS, Schmidt EW. 2015. Species specificity of symbiosis and secondary metabolism in ascidians. ISME J 9:615–628. doi:10.1038/ismej.2014.152 25171330 PMC4331574

[109] López-Legentil S, Erwin PM, Turon M, Yarden O. 2015. Diversity of fungi isolated from three temperate ascidians. Symbiosis 66:99–106. doi:10.1007/s13199-015-0339-x

[110] MacIntyre LW, Marchbank DH, Correa H, Kerr RG. 2018. Fusaristatin C, a cyclic lipodepsipeptide from Pithomyces sp. RKDO 1698. J Nat Prod 81:2768–2772. doi:10.1021/acs.jnatprod.8b00787 30525612

[111] Kobayashi J, Kubota T, Ishiguro Y, Yamamoto S, Fromont J. 2010. Platisidines A-C, N-methylpyridinium alkaloids from an Okinawan marine sponge of plakortis species. Heterocycles 80:1407. doi:10.3987/COM-09-S(S)131

[112] Wu J, Wang P, Zhang D, Chen S, Sun Y, Wu J. 2016. Catalysis of oxygen reduction reaction by an iron-reducing bacterium isolated from marine corrosion product layers. J Electroanal Chem (Lausanne) 774:83–87. doi:10.1016/j.jelechem.2016.04.053

[113] Xia Y, Liu S, Li C, Ai Z, Shen W, Ren W, Yang X. 2020. Discovery of a novel ferroptosis inducer-talaroconvolutin A-killing colorectal cancer cells in vitro and in vivo*.* Cell Death Dis 11:988. doi:10.1038/s41419-020-03194-2 33203867 PMC7673992

[114] Baar HJW, Roche J. 2003. Trace metals in the oceans: evolution, biology and global change, p 79–105. In Wefer G, Lamy F, Mantoura F (ed), Marine science frontiers for Europe. Springer, Berlin.

[115] Nakagawa S, Saito H, Tame A, Hirai M, Yamaguchi H, Sunata T, Aida M, Muto H, Sawayama S, Takaki Y. 2017. Microbiota in the coelomic fluid of two common coastal starfish species and characterization of an abundant helicobacter-related taxon. Sci Rep 7:8764. doi:10.1038/s41598-017-09355-2 28821872 PMC5562702

[116] Beinart RA, Luo C, Konstantinidis KT, Stewart FJ, Girguis PR. 2019. The bacterial symbionts of closely related hydrothermal vent snails with distinct geochemical habitats show broad similarity in chemoautotrophic gene content. Front Microbiol 10:1818. doi:10.3389/fmicb.2019.01818 31474946 PMC6702916

[117] Ballarin L, Cima F, Sabbadin A. 1995. Morula cells and histocompatibility in the colonial ascidian Botryllus schlosseri*.* Zool Sci 12:757–764. doi:10.2108/zsj.12.757

[118] Dingley AL, Kustin K, Macara IG, McLeod GC. 1981. Accumulation of vanadium by tunicate blood cells occurs via a specific anion transport system. Biochim Biophys Acta 649:493–502. doi:10.1016/0005-2736(81)90152-8

[119] Michibata H, Ueki T. 2012. High levels of vanadium in ascidians, p 51–71. In Michibata H (ed), Vanadium: biochemical and molecular biological approaches. Springer Netherlands, Dordrecht.

[120] Odate S, Pawlik JR. 2007. The role of vanadium in the chemical defense of the solitary tunicate, Phallusia nigra. J Chem Ecol 33:643–654. doi:10.1007/s10886-007-9251-z 17265174

[121] Ueki T. 2016. Bioaccumulation of vanadium by vanadium-resistant bacteria isolated from the intestine of Ascidia sydneiensis samea. Mar Biotechnol 18:359–371. doi:10.1007/s10126-016-9697-5 27177911

[122] Ueki T, Fujie M, Satoh N. 2019. Symbiotic bacteria associated with ascidian vanadium accumulation identified by 16S rRNA amplicon sequencing. Mar Genomics 43:33–42. doi:10.1016/j.margen.2018.10.006 30420273

[123] Ku C, Barak-Gavish N, Maienschein-Cline M, Green SJ, Vardi A. 2018. Complete genome sequence of Sulfitobacter sp. Strain D7, a virulent bacterium isolated from an Emiliania huxleyi algal bloom in the North Atlantic. Microbiol Resour Announc 7:e01379-18. doi:10.1128/MRA.01379-18 30533799 PMC6256486

[124] Porcheron G, Garénaux A, Proulx J, Sabri M, Dozois CM. 2013. Iron, copper, zinc, and manganese transport and regulation in pathogenic Enterobacteria: correlations between strains, site of infection and the relative importance of the different metal transport systems for virulence. Front Cell Infect Microbiol 3:90. doi:10.3389/fcimb.2013.00090 24367764 PMC3852070

[125] Hood MI, Skaar EP. 2012. Nutritional immunity: transition metals at the pathogen-host interface. Nat Rev Microbiol 10:525–537. doi:10.1038/nrmicro2836 22796883 PMC3875331

[126] Murdoch CC, Skaar EP. 2022. Nutritional immunity: the battle for nutrient metals at the host-pathogen interface. Nat Rev Microbiol 20:657–670. doi:10.1038/s41579-022-00745-6 35641670 PMC9153222

[127] Martin JE, Le MT, Bhattarai N, Capdevila DA, Shen J, Winkler ME, Giedroc DP. 2019. A Mn-sensing riboswitch activates expression of a Mn^2+^/Ca^2+^ ATPase transporter in Streptococcus. Nucleic Acids Res 47:6885–6899. doi:10.1093/nar/gkz494 31165873 PMC6649816

[128] Sousa Gerós A, Simmons A, Drakesmith H, Aulicino A, Frost JN. 2020. The battle for iron in enteric infections. Immunology 161:186–199. doi:10.1111/imm.13236 32639029 PMC7576875

[129] Monteith AJ, Skaar EP. 2021. The impact of metal availability on immune function during infection. Trends Endocrinol Metab 32:916–928. doi:10.1016/j.tem.2021.08.004 34483037 PMC8516721

